# The clinical and cost effectiveness of a conservative treatment pathway compared to appendicectomy in children with uncomplicated acute appendicitis: study protocol for a randomised controlled multi-centre open-label parallel-group non-inferiority trial (CONTRACT 2 trial) in the United Kingdom (UK)

**DOI:** 10.1186/s13063-025-09282-y

**Published:** 2025-12-04

**Authors:** Natalia Vadimovna Permyakova, Isabel C Reading, Young Bridget, Simon Eaton, Maria Chorozoglou, Frances C Sherratt, Harriet Corbett, Darran Ball, Andrew Cook, Jessica Kelly, Elizabeth Dixon, Nigel J Hall

**Affiliations:** 1 Southampton Clinical Trials Unit, Faculty of Medicine, University of Southampton, Southampton, UK; 2 Primary Care and Population Sciences, Faculty of Medicine, University of Southampton, Southampton, UK; 3 Department of Public Health, Policy and Systems, University of Liverpool, Liverpool, UK; 4 UCL Great Ormond Street Institute of Child Health, London, UK; 5 Southampton Health Technology Assessment Centre, Faculty of Medicine, University of Southampton, Southampton, UK; 6 Department of Psychological Sciences, University of Liverpool, Liverpool, UK; 7 Department of Surgery, Alder Hey Children’s NHS Foundation Trust, Liverpool, UK; 8 University Surgery Unit, Faculty of Medicine, University of Southampton, Southampton, UK

**Keywords:** Appendicitis, Appendicectomy, Non-operative treatment, Abdominal pain, Paediatric surgery, Randomised controlled trial, Non-inferiority, Evidence-based medicine, Qualitative research, Economic evaluation

## Abstract

**Background:**

Currently, the most frequently used treatment for acute appendicitis in children in the United Kingdom (UK) is an appendicectomy. However, there is increasing scientific and patient interest and research into non-operative treatment of appendicitis. Despite a number of non-randomised studies in children and randomised studies in adults, comparative outcomes of non-operative treatment and appendicectomy in comparable groups of children remain unknown. Following the successful completion of a feasibility study, we now aim to perform a UK-based multi-centre open-label randomised controlled trial (RCT) to investigate the clinical and cost-effectiveness of non-operative treatment pathway of acute uncomplicated appendicitis in children compared with appendicectomy.

**Methods:**

Non-inferiority RCT with internal pilot, health economic evaluation and qualitative communication sub-study. The study is conducted in England, Northern Ireland, Scotland and Wales at both specialist children’s hospitals and district general hospitals. Children (aged 4–15 years inclusive) diagnosed with acute uncomplicated appendicitis that would normally be treated with an appendicectomy are eligible for the RCT. Exclusion criteria include clinical/radiological suspicion of perforated appendicitis, appendix mass or previous non-operative treatment of appendicitis. Sample size is 376 participants, recruited by surgeons and supported by research staff and randomised with a 1:1 allocation ratio to either non-operative treatment pathway (intervention) or appendicectomy (control). Participants in the intervention arm are treated with antibiotics, analgesia and regular clinical assessment to ensure clinical improvement. Participants in the control arm receive appendicectomy. Randomisation is minimised by age, sex, duration of symptoms and centre. The primary end-point is a composite outcome of treatment success at 1 year following randomisation. Secondary outcomes include: duration of hospital stay, measures of recovery from acute appendicitis, complications, need for further treatment, persistent symptoms, health care resource use, quality of life and costs. Adverse events, serious adverse events and suspected unexpected serious adverse events are collected directly on the database and by paper form up to 12-month visit. Primary outcome will be analysed on a non-inferiority basis using a 20% non-inferiority margin to test the hypothesis that non-operative treatment pathway is non-inferior to appendicectomy. Children and families who are approached for the RCT will be invited to participate in the embedded qualitative sub-study. This will include recording of recruitment consultations, which will inform future interventions to optimise recruitment. We have involved children, young people and parents in study design and delivery.

**Discussion:**

This RCT will allow determination of the comparative clinical and cost-effectiveness of non-operative treatment pathway compared to appendicectomy for children with uncomplicated acute appendicitis in the UK.

**Trial status:**

First planned enrolment—December 2022, first actual recruit—March 2022, current status of trial—open to recruitment.

**Trial registration:**

ISRCTN16720026. Registered on July 28, 2021.

## Administrative information

**Table:** 

Title {1}	The clinical and cost effectiveness of a conservative treatment compared to appendicectomy in children with uncomplicated acute appendicitis: study protocol for a randomised controlled multi-centre open-label parallel-group non-inferiority trial (CONTRACT 2 trial) in the United Kingdom (UK)
Trial registration {2a and 2b}	ISRCTN16720026. Date of registration: 28th July 2021
Protocol version {3}	Version 8.0 from 30-OCT-2024
Funding {4}	This Trial is primarily funded by NIHR Health Technology Assessment programme, project number NIHR131346
Author details {5a}	Permyakova, Natalia V; Southampton Clinical Trials Unit, Faculty of Medicine, University of Southampton, Southampton, UK Reading, Isabel C; Primary Care and Population Sciences, Faculty of Medicine, University of Southampton, Southampton, UK Young, Bridget; Department of Public Health, Policy and Systems, University of Liverpool, Liverpool, UK Eaton, Simon; UCL Great Ormond Street Institute of Child Health, London, UK Chorozoglou, Maria; Southampton Health Technology Assessment Centre, Faculty of Medicine, University of Southampton, Southampton, UK Sherratt Frances C; Department of Psychological Sciences, University of Liverpool, Liverpool, UK Corbett, Harriet; Department of Surgery, Alder Hey Children’s NHS Foundation Trust, Liverpool, UK Ball, Darran; c/o Southampton Clinical Trials Unit, Faculty of Medicine, University of Southampton, Southampton, UK Cook, Andrew; Southampton Clinical Trials Unit, Faculty of Medicine, University of Southampton, Southampton, UK Kelly, Jessica; Southampton Clinical Trials Unit, Faculty of Medicine, University of Southampton, Southampton, UK Dixon, Elizabeth; Southampton Clinical Trials Unit, Faculty of Medicine, University of Southampton, Southampton, UK Hall, Nigel J; University Surgery Unit, Faculty of Medicine, University of Southampton, Southampton, UK
Name and contact information for the trial sponsor {5b}	University Hospitals Southampton NHS Foundation Trust SGH—Level E, Laboratory & Pathology Block, SCBR, LE123—MP 138 Southampton SO16 6YD United Kingdom (0)23 8120 5662 sponsor@uhs.nhs.uk
Role of sponsor {5c}	Sponsor has delegated responsibility in trial design; collection, management, analysis and interpretation of data; writing of the report; and the decision to submit the report for publication to the trial team at Southampton Clinical Trials Unit

## Introduction

### Background and rationale {6a}

Acute appendicitis is the most common surgical emergency in children [1]. The lifetime risk is 7–8% with a peak incidence in the early teens [2, 3]. Appendicectomy is considered the gold standard treatment by most surgeons. In 2023–2024, there were 7408 emergency appendicectomies in England in children aged < 16 years old [4]. Due to this volume, management of children with appendicitis incurs significant costs for the healthcare service each year [5].

For parents, the need for emergency surgery is frightening and many are keen to avoid surgery if an alternative is available. Surgeons are frequently asked ‘Does my child really need an operation?’ Whilst appendicectomy is generally safe, it involves a general anaesthetic and an abdominal operation with inherent risks and potential complications. Work we have undertaken with parents shows that over 80% would be willing to consider non-operative treatment pathway for their child with appendicitis, and 60% would prefer non-operative treatment to surgery if outcomes were similar (20% preferred surgery and 20% expressed no-preference) [6].

An alternative would be to treat children non-operatively, without surgery but with antibiotics. This would have the benefit of avoiding an operation and potential side effects but would only be acceptable if antibiotic treatment has a high success rate and the risk of serious complications and recurrent appendicitis is low. Although research to date has confirmed that most children with uncomplicated acute appendicitis can be successfully treated without surgery [7–12], there have been no adequately powered randomised controlled trials (RCTs) reported before the start of CONTRACT 2 that directly compare these two very different treatments and conduct an economic evaluation. Comparative data are needed to help inform future practice and, in particular, to inform treatment choices by children, parents, clinicians, National Health Service (NHS) commissioners and healthcare policy makers.

Anticipating challenges in recruiting to such an RCT, we have already successfully performed a detailed feasibility study with embedded qualitative research and work to inform a health economic analysis [5, 13–19]. From this, we have confirmed that we can recruit participants to an RCT, gained valuable insights to optimise trial recruitment and retention, confirmed the safety of a non-operative treatment pathway in a UK setting, determined outcomes of importance to measure, discussed the acceptable non-inferiority margin with clinicians, patients and parents [13, 16, 19] and determined cost-drivers to underpin the design of a full economic analysis [5, 15]. We now plan to perform a large multi-centre non-inferiority RCT, generating the evidence to inform future clinical practice in the UK.

### Objectives {7}

Primary: To determine whether a non-operative treatment pathway is non-inferior to appendicectomy for the treatment of children with uncomplicated acute appendicitis. The primary outcome of ‘treatment success’ will be assessed at 1 year following randomisation.

Secondary: To compare a non-operative treatment pathway with appendicectomy in terms of other important patient- and family-centred outcomes and cost. Outcomes will include duration of hospital stay, measures of recovery from acute appendicitis, complications, need for further treatment, persistent symptoms, health care resource use, quality of life and costs. We will measure all applicable outcomes in our developed core outcome set [13, 19].

### Trial design {8}

**Fig. 1:**
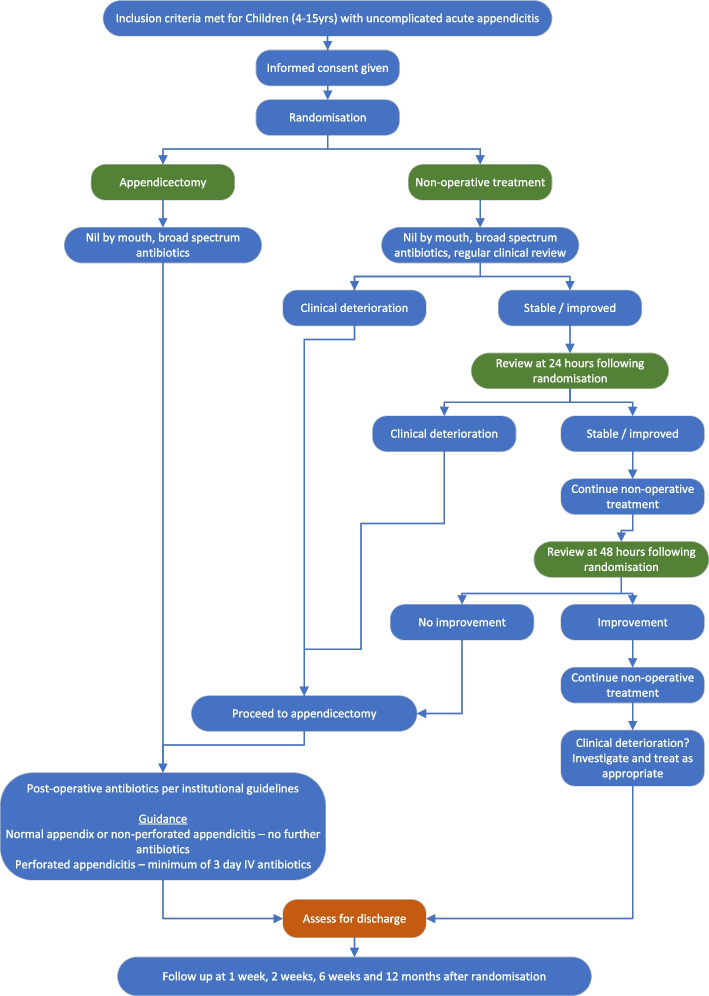
Trial schema

A multi-centre, open-label, parallel-group, non-inferiority randomised controlled trial comparing a non-operative treatment pathway with appendicectomy with internal pilot, health economic evaluation and qualitative sub-study. Both groups of children will receive broad spectrum antibiotics from the point of diagnosis; one group of children will undergo urgent appendicectomy, the other will be treated non-operatively with continuation of broad spectrum intravenous antibiotics. Patients enrolled in the study will be randomised at a ratio of 1:1. See Fig. 1 for a Trial schema overview.

#### Internal pilot stage

The pilot stage is embedded in this RCT to ensure that recruitment outside of the three sites used in our feasibility study is possible, confirm our anticipated recruitment rate, implement recruiter training and adjust the overall trial profile if necessary. We have devised a comprehensive program of recruiter training and re-training, using an evidence-based approach with some limited qualitative work and informed by our feasibility study [5, 13–19], which we believe is crucial to achieving target recruitment.

We have focused pilot stage metrics on actual recruitment, which is dependent on initiating sites and successfully recruiting participants. In our feasibility study [5, 13–19], we successfully opened all three sites on the same day, whilst in CONTRACT 2 we stagger site opening and aim to open all sites within the first 6 months of recruitment.

Internal pilot data will be analysed after 12 months from the start of recruitment to assess trial and recruitment progress. The decision of progression to full trial will be taken by the Trial Steering Committee (TSC) in conjunction with the funder, based on traffic light criteria assessed after 12 months of recruitment (Table 1).

**Table 1:** Traffic light criteria for the pilot phase assessed after 12 months of recruitment

Pilot phase criteria	Red	Amber	Green
% Threshold (of participants)	<75%	75-99%	100%
Total number of participants recruited	<120	120-159	≥160
Number of sited opened	<7	7-9	≥10

Note: Green - immediate progression to full RCT; Amber - prolongation of pilot phase for further 6 months to allow further identification of sites and/or further training at existing sites; Red - undertake urgent detailed review of options with TSC and report to HTA

All data from the internal pilot and subsequent RCT will be used in the final analysis. No interim analysis of effectiveness is planned. Data will be presented according to the Consolidated Standards of Reporting Trials (CONSORT) guidelines extension for non-inferiority trials, 2012 [20].

## Methods: participants, interventions and outcomes

### Study setting {9}

Specialist Paediatric Surgical Units and General Hospitals in the UK.

### Eligibility criteria {10}

Eligible participants will be identified by the clinical team at time of diagnosis of acute appendicitis.

**Inclusion criteria**:

1. Children aged 4–15 years
2. Clinical diagnosis, with or without radiological assessment, of acute appendicitis which prior to study commencement would be treated with appendicectomy
3. Written informed parental or guardian consent, with child assent if appropriate

**Exclusion criteria**:

1. Complicated appendicitis score (Table 2) of 4 or greater
2. Clinical or Radiological findings to suggest perforated appendicitis
3. Presentation with appendix mass
4. Previous episode of appendicitis or appendix mass treated non-operatively
5. Major anaesthetic risk precluding allocation to the appendicectomy arm
6. Documented allergy to first and second line broad spectrum antibiotics preventing allocation to non-operative treatment arm
7. Positive pregnancy test (only required for female patients of child bearing potential)

**Table 2:** Complicated appendicitis score

Parameter	Points
Rebound tenderness	1
Duration of pain ≥ 48 h	1
Temperature* ≥ 37.5 °C	1
Neutrophil count ≥ 11 (× 10^9^/L)	2
CRP ≥ 50 (mg/L)	2

*Documented temperature, in hospital, at any stage prior to diagnosis; *CRP* - C-reactive protein

Note: The complicated appendicitis score was developed following feedback from surgeons during our feasibility study [5, 13–19] indicating that a more objective means of distinguishing between children with uncomplicated appendicitis and those with more advanced disease was necessary

### Who will take informed consent? {26a}

Recruitment discussions will be conducted by the clinical team with responsibility for the patient, and consent will be taken by clinical teams or research nurses.

### Additional consent provisions for collection and use of participant data and biological specimens {26b}

We will seek consent from parents to contact them at yearly intervals by telephone/e-mail for longer term follow-up, up to a maximum of 5 years. We will request consent to store personal data (telephone number and e-mail address) securely for the purposes of this RCT only. We will also seek consent to use a patient identifier for the purposes of recording long term outcomes from national datasets of routinely collected data. These activities are outside of the current funding remit.

## Interventions

### Explanation for the choice of comparators {6b}

Currently, the most frequently used treatment for children with appendicitis in the UK is appendicectomy. There is increasing interest and demand for non-operative treatment of uncomplicated appendicitis in children but a lack of data comparing non-operative treatment pathway with appendicectomy. Comparative data are required to inform clinical practice and shared decision making.

### Intervention description {11a}

#### Non-operative treatment group

This treatment pathway will comprise (i) fluid resuscitation, (ii) a minimum of 24-h intravenous antibiotics (determined by local hospital standard antibiotics for appendicitis, as per our feasibility study), (iii) analgesia and (iv) regular clinical review to detect symptoms and signs of significant clinical deterioration including, but not limited to, increasing fever, increasing tachycardia and increasing pain/tenderness. In our feasibility study [5, 13–19], we recommended a minimum period of being ‘nil-by-mouth’ but learnt from experience that this was unnecessary and deterred some children from participating, therefore this requirement has been removed for this RCT.

Once children have received minimum 24-h intravenous antibiotics, have been afebrile for 24 h and are tolerating oral intake, they will be converted to oral antibiotics (co-amoxiclav as per standard practice). Once discharge criteria are met (see below), they will be discharged. In total, children in the intervention group will receive a course of 10 days antibiotics following randomisation. Children who receive non-operative treatment will not be offered appendicectomy, but they and their parents will be counselled about the risk of recurrence and appropriate action to take. Children who require appendicectomy for failure of non-operative treatment during the initial admission will be treated post-operatively according to a standardised treatment regime already in use at participating institutions, and identical to that to be used in children in the appendicectomy group.

#### Appendicectomy group

Children allocated to appendicectomy group will undergo urgent appendicectomy according to current treatment pathways at participating centres. The procedure may be performed by a suitably experienced trainee (as is routine current practice) or a consultant. The procedure may be performed open or laparoscopically at the discretion of the clinical team according to their current practice (in our feasibility study, open appendicectomy was a rare event).

It is recommended to take a peritoneal microbiology swab at the time the peritoneum is first opened and any peritoneal fluid sent for microbiological culture. Participants will receive intravenous antibiotics from the time of randomisation and be treated post-operatively with intravenous antibiotics (according to existing treatment pathways already in use at participating centres). Typically, children with uncomplicated acute appendicitis or a macroscopically normal appendix will receive no further antibiotics. If unexpectedly a perforated appendix is discovered at surgery (defined as a faecalith or faecal matter within the peritoneal cavity or visualisation of a hole in the appendix) [21], then intravenous antibiotics will be continued for a minimum of 3 days, with a minimum total course of antibiotics of 5 days (intravenous and oral). It is not possible to completely ‘protocolise’ the duration of antibiotic therapy due to anticipated variation in response to treatment. Children with perforated appendix will remain in the RCT.

The type of antibiotics initially used will be according to current standard local hospital policy. Any child failing to respond to these first line antibiotics will be treated as is clinically appropriate with a longer course of antibiotics, or a change in antibiotic therapy with choice of antibiotic determined by intra-operative swab or fluid culture. Post-operatively, children with uncomplicated acute appendicitis or a normal appendix will not routinely have a nasogastric tube, nor a urinary catheter; they will receive oral intake as tolerated after surgery.

#### Discharge from hospital

The decision to discharge the child home will be made by the clinical team using standard clinical criteria for both treatment groups which will be (i) afebrile, (ii) vital signs within normal limits, (iii) able to tolerate oral intake and (iv) able to mobilise. The time the decision to discharge was made and the time of actual discharge will be recorded. Prior to discharge, parents will be assisted to download a smartphone app to allow daily data collection directly from parents during the first 4 weeks following hospital discharge. A paper patient diary card can be supplied for those without access to a smartphone.

All participants, across both treatment groups, will be provided with a discharge pack. This pack will contain a leaflet highlighting concerning symptoms and action to be taken should any of them occur, including advice to contact a member of the medical team at each participating hospital (with relevant contact details), or the participant’s GP in an emergency and the telephone number of the research nursing team at each site for less urgent concerns. We will write to the participant’s GP to inform them of their patient’s inclusion in the RCT.

### Criteria for discontinuing or modifying allocated interventions {11b}

Children receiving non-operative treatment who, in the opinion of the consultant surgeon in charge of their care, have clinically deteriorated such that immediate appendicectomy is mandated, will undergo appendicectomy at any stage. A formal review will be performed at 24 h following randomisation, and any child deemed to have significantly deteriorated (e.g. deterioration in objective clinical observations which in the view of the surgeon in charge of their care justifies urgent appendicectomy) will undergo appendicectomy. Those who are stable or clinically improving will continue with non-operative treatment. Those who are not showing clinical signs of improvement at 48 h following randomisation will undergo appendicectomy. This decision will be made based on the clinical judgement of the treating consultant as is current practice rather than on any predefined set of criteria for which evidence does not currently exist. These decision points are included in the non-operative treatment pathway and therefore these patients will have been considered to have received their allocated intervention.

### Strategies to improve adherence to interventions {11c}

Both interventions are routine clinical interventions in the acute care setting. We do not therefore anticipate significant challenges with adherence but will monitor and address this through site training if necessary. Site staff will check the database regularly and contact any patients who have not provided follow-up data as a prompt to complete.

### Relevant concomitant care permitted or prohibited during the trial {11d}

None for this trial.

### Provisions for post-trial care {30}

All participants will be followed up for 1 year following randomisation. Formal follow-up appointments will take place at 6 weeks, 4 months, 8 months and 12 months following randomisation. The 6-week follow-up appointment will take place in the outpatient clinic or Clinical Research Facility at each participating centre (can be also completed via a phone call in case of patient’s unavailability for an in-person appointment, e.g. due to Covid). The 4-, 8- and 12-month appointments should be completed over the phone or video call. The follow-up appointments should be completed no earlier than 1 week before the projected visit date, and no later than 2 weeks after the projected visit date.

Data will be collected during these follow-up visits to ensure high accuracy. These visits will ensure completeness of the dataset collected, in particular time to return to daily activities, recurrent appendix-related problems (including unexplained abdominal pain and recurrence) and resource use data. If the visit is missed, a nurse will access the patient’s hospital records to obtain necessary information for the primary outcome of this RCT: any re-admissions and/or complications since the last visit.

Children treated non-operatively who present with suspected recurrent appendicitis during the 1-year follow-up period will not be eligible for re-randomisation. These cases will be recorded in this RCT as a failure of non-operative treatment pathway. These cases are typically treated with appendicectomy and not treated non-operatively, unless re-presentation is with appendix mass or abscess and non-operative treatment is felt preferable by the clinical team.

Children treated with appendicectomy who present with a complication related to their initial procedure shall be treated as deemed clinically appropriate by the clinical team.

### Outcomes {12}

Primary outcome is treatment success (binary variable ‘yes/no’), to be measured at 1 year following randomisation and defined as recovery from acute appendicitis and having none of the following: (i) negative appendicectomy (no histological evidence of inflammation), (ii) complication requiring intervention under general anaesthesia, (iii) failure of non-operative treatment pathway during initial hospital admission (treated with appendicectomy) and (iv) recurrent appendicitis (based on histological assessment or further episode of non-operative treatment if appendicectomy not performed). Secondary outcomes and measurement timepoints are listed in Table 3 below.

**Table 3:** Secondary outcomes

Outcome (Y/N, otherwise stated)	Timing of measurement	Method of measurement
Negative appendicectomy*^$^	Hospital discharge, 6-week review	Research nurse
Intra-abdominal abscess*	Hospital discharge, 6-week review	Research nurse
Reoperation*^$^	Hospital discharge, 6-week, 4-, 8-, 12-month	Research nurse
Bowel obstruction*	Hospital discharge, 6-week, 4-, 8-, 12- month	Research nurse
Wound infection*	Hospital discharge, 6-week review	Research nurse
Other wound complication*	Hospital discharge, 6-week review	Research nurse
Antibiotic failure*^$^	Hospital discharge, 6-week review	Research nurse
Length of hospital stay* [days]	Hospital discharge, 6-week, 4-, 8-, 12-month	Research nurse
Histology of appendix^1^	Hospital discharge, 6-week, 4-, 8-, 12-month	Research nurse
Adverse events*	Hospital discharge, 6-week, 4-, 8-, 12-month	Research nurse
Recurrent appendicitis*^$+^	6-week and 4-, 8-, 12-month	Research nurse
Readmission to hospital*	6-week and 4-, 8-, 12-month	Research nurse
Appendicectomy without recurrent appendicitis on histology	6-week and 4-, 8-, 12-month	Research nurse
Caregiver and patient’s quality of life* (CHU-9D)^2^	At randomisation, week 1–4, week 6 and 4-, 8-, 12-month	Research Nurse (option of smartphone app at weeks 1–4)
Healthcare resource use (shortened CSRI)^3^	6-week and 4-, 8-, 12-month	Research nurse
Death*	Hospital discharge, 6-week and 4-, 8-, 12-month	Research nurse
Pain meds taken for appendicitis	Hospital Discharge, then daily for 3 weeks	Smartphone app/paper card
Able to do normal daily activities	Hospital Discharge, then daily for 3 weeks	Smartphone app/paper card
Attended school (if applicable)	Hospital Discharge, then daily for 3 weeks	Smartphone app/paper card
Able to do full activities*	Hospital Discharge, then daily for 3 weeks	Smartphone app/paper card
Parent/main caregiver missed work	Hospital Discharge, then daily for 3 weeks	Smartphone app/paper card

*Indicates outcome within core outcome set

$ Indicates part of composite primary outcome

^+^Recurrent appendicitis is defined as symptom recurrence followed by EITHER appendicectomy with histological confirmation of acute appendicitis OR a clinician diagnosis of appendicitis with appendix mass or abscess treated non-operatively; appendicectomy without histological confirmation of acute appendicitis is a separate secondary outcome

^1^Histology of appendix consists of the next categories: (1) not applicable, (2) no abnormality, (3) simple acute appendicitis, (4) perforated, (5) Enterobius detected, (6) other

^2^For the purposes of economic evaluations, a utility score (ranging from 0 to 1, with 1 being perfect health) is calculated as the sum of the scores for each of nine dimensions of the CHU-9D questionnaire (worry, sadness, pain, tiredness, annoyance, schoolwork/homework, sleep, daily routine and ability to join in activities)

^3^A short modified version of the CSRI (Client Service Resource Inventory) questionnaire collects the information in the last 6 weeks on: (1) health and social care service use (the number of hospital stays, visits to a health care professionals and the types of health services), (2) family born costs (expenses related to child’s appendicitis, e.g., travel to hospital, painkillers, heat or massage oils, herbal or complimentary remedies), (3) parent’s, carer’s, partner’s employment loss and child’s absent from school (if yes, number of days)

#### Embedded health economic study

Data generated from our feasibility study [5, 13–19] provided important insights to optimise the design of the economic evaluation in this definitive RCT. This includes selection of the most appropriate Health-related quality of life (HRQoL) instrument, as well as defining the most appropriate methods and data collection tools. The economic analysis of this study will be based on an assessment of the incremental cost per successfully treated child (primary outcome) and the incremental cost per Quality Adjusted Life Year (QALY) gained.

In our feasibility study, following a detailed micro-costing approach, we established the potential of economic savings for the NHS [5, 15]. Alongside this RCT, we will conduct a full economic evaluation assessing the cost-effectiveness of the non-operative treatment pathway compared to appendicectomy. Therefore, the within trial economic analysis, adherent to guidelines for good economic evaluation practice [22–24], will include (i) a cost-effectiveness analysis (CEA) using the primary outcome at the end of the follow-up (1 year) and (ii) a cost-utility analysis (CUA) using HRQoL (caregiver-reported CHU-9D) collected at the 6-week visit to assess the short-term impact of the intervention in terms of QALYs.

The perspective of the economic study will be that of the NHS. All cost-effectiveness results will be presented on (i) the cost-effectiveness plane, which captures the uncertainty around the results showing the incremental costs and incremental effects of the comparison of interest in a 2-dimentional plot, and (ii) the cost-effectiveness acceptability curves, which graphically represent the uncertainty in terms of probabilities, regarding the cost-effectiveness of the intervention. We will also report on the economic implications for the NHS of non-operative treatment pathway. This will include developing and reporting a tariff for the non-operative treatment of uncomplicated acute appendicitis since this is not currently available within the NHS Reference Costs data.

### Data collection and health economic outcomes

#### Resource use, costs and HRQoL data

In accordance with findings of our feasibility study [5, 13–19], the primary care data from parents will be collected by research nurses during phone calls and in person interviews using a short modified version of the CSRI (Client Service Resource Inventory) questionnaire [25–27]. For secondary care data, we will use the Patient Level Information and Costing Systems (PLICS) [28] data for acute services records activity and cost information for admitted patient care, outpatient appointments and A&E attendances. The results from our feasibility study support the use of PLICS data as a reliable alternative to micro-costing [5]. Unit cost will use the NHS Reference Costs [29] and Personal Social Services Research Unit (PSSRU) [30] data in addition to other unit costs as appropriate. To allow reporting in QALY terms, we will use the caregiver-reported CHU-9D.

#### Timing of health economic data collection

The data collection refers to hospital records for admission(s) to hospital(s) up to 12-month duration, including admissions for recurrent appendicitis and/or other related admissions. Data regarding health care resource use will be collected directly from parents/guardians through a smartphone app weekly up to 4 weeks following randomisation. These records will be discussed in detail during a face-to-face visit at 6 weeks. Health economics (HE) data will be collected during phone calls at 4-, 8- and 12-month visits, and the e-CSRI questionnaire will be completed by a research nurse during these visits at 6 weeks and 4, 8 and 12 months.

Our previous work revealed that timing is an important consideration for collecting QoL data and estimating QALYs for this intervention. While there was a significant difference in QoL at 2 weeks, this difference was not present at 6 weeks, by which time both groups had returned to almost full health. Therefore, we will record QoL data at baseline, weekly up to 4 weeks and then at 6 weeks, in order to identify the return to full health and normal life for children in both arms. The CUA will report these short-term outcomes in QALYs.

### Participant timeline {13}

Table 4 shows the participant timeline for patient’s observations and procedures.

**Table 4:** Schedule of observations and procedures

**Visit/time point (all measured from randomisation:**	**Baseline/randomisation**	**Treatment**	**Dis-charge**	**1 week**^**a**^ (+ 3 days)	**2 weeks**^**a**^ (+ 3 days)	**3 weeks**^**a**^ (+ 3 days)	**4 weeks**^**a**^ (+ 3 days)	**6 weeks** (− 1 week/+ 2 weeks)	**4 months**^**a**^ (− 1 week/+ 2 weeks)	**8 months**^**a**^ (− 1 week/+ 2 weeks)	**12 months**^**a**^ (− 1 week/+ 2 weeks)
Informed consent	**X**										
Eligibility evaluation	**X**										
Medical history	**X**										
Diagnostic tests as per standard practice (blood test—Total WBC/CRP/Neutrophil, CT scan, Ultrasound)	**X**										
Pregnancy test^b^	**X**										
Physical exam (abdomen exam)								**X**			
Vital signs (temperature)	**X**										
Randomisation	**X**										
Appendicectomy (arm B only)		**X**									
IV antibiotics (arm A only)		**X**									
Doctor/healthcare professional assessment	**X**							**X**			
Histology following surgery								**X**	**X**	**X**	**X**
Discharge assessment			**X**								
Adverse Events	**X**		**X**	**X**	**X**	**X**	**X**	**X**	**X**	**X**	**X**
Health economics—resource use	**X**		**X**	**X**	**X**	**X**	**X**	**X**	**X**	**X**	**X**
CHU-9D	**X**			**X**	**X**	**X**	**X**	**X**	**X**	**X**	**X**
Patient data collected on app				**X**	**X**	**X**	**X**				
Client service receipt inventory (CSRI)								**X**	**X**	**X**	**X**
Recurrence								**X**	**X**	**X**	**X**
Complication requiring intervention under general anaesthesia	**x**							**x**	**x**	**x**	**x**

^a^To be collected remotely via phone call and/or app

Data collected on app:

▪ Pain relief taken Y/N

▪ CHU 9D proxy and self-report where appropriate

▪ Antibiotics taken Y/N

▪ Able to do normal daily activities Y/N

▪ Attended school Y/N

▪ Able to do full activities Y/N

▪ Parents missed work Y/N

▪ The last week have you had any contact with a health care professional(s) for your child’s appendicitis? Y/N

^b^Only for those of childbearing potential

NB: The Participant/legal representative is free to withdraw consent at any time without providing a reason. When withdrawn, the participant will continue to receive standard clinical care. Follow up data will continue to be collected (unless the participant/legal representative has specifically stated that they do not want this to happen)

### Sample size {14}

We will test the hypothesis that non-operative treatment pathway is non-inferior to appendicectomy. We anticipate a difference between the treatment arms with respect to the primary outcome of 8 percentage points (assuming 88% treatment success in the appendicectomy group and 80% in the non-operative group). Since there are potential further benefits to non-operative treatment that are not included within the primary outcome (e.g. greater parental acceptability, avoidance of general anaesthesia, avoidance of surgical complications, cost), it is appropriate to use a wider non-inferiority margin for our analysis. The size of this non-inferiority margin was explored with both surgeons and parents in our feasibility study [5, 13–19], which was pre-specified at 20% for this trial. Of interest, this is the same margin that was defined by a Cochrane review as being ‘clinically relevant’ [31].

Given that the assumed difference in success rate is non-zero (8%), a one-sided 5% significance level was deemed appropriate. Based on the estimates of non-zero assumed difference of 8% and non-inferiority margin of 20%, with a one-sided 5% significance level and 90% power, this trial will require a total of 318 cases analysed at 1 year (159 per arm). Allowing for 15% loss to follow up, the recruitment target will be 376 participants (188 per arm).

### Recruitment {15}

Recruitment will be performed by surgeons and supported by research nurses since parents do not feel it is appropriate to be recruited into this trial by anyone other than a surgeon, as it was found in our preparatory work with the National Institute of Health Research Clinical Research Network (NIHR CRN) (Children) Young Person’s Advisory Groups (YPAGs). The CRN has also indicated that they do not think it is appropriate for nurses to recruit to this study alone due to the nature of the intervention which will challenge commonly-held beliefs about appendicectomy as best treatment for appendicitis, and the relatively short timeframe necessary for a decision to be made. Therefore, we will utilise members of the clinical team (Specialist Surgical Trainees and Consultants) to recruit patients to the study in conjunction with research nurses. Recruitment capacity will therefore be available 24 h per day.

Parents will be approached by a member of the surgical team (with or without a research nurse) who will explain the study to them and invite them to participate. Prior to this discussion, verbal permission will be taken for the recruitment discussion to be voice-recorded. The CONTRACT 2 trial will be explained to parents and children with the aid of age-specific information sheets and a short video presentation. The patient video will also be made available via a web-link to allow parents/guardians, who cannot be in hospital with their children at the time of recruitment, to access the same trial information as the consenting parent/guardian.

We will provide an educational package to clinical staff at each centre. This will include (i) educational evenings at or near each centre (or delivered remotely via videoconference) to which all members of the clinical team will be invited (core and specialist surgical trainees, research nurses and consultant surgeons); (ii) a short video to be shown to potential participants during the recruitment process; and (iii) age-appropriate participant information sheet and consent form.

#### Embedded communication sub-study

A qualitative sub-study is embedded during the pilot phase of this RCT with an aim to identify potential barriers to recruitment and enhance informed consent. This communication sub-study was designed learning from our experience in our feasibility study [5, 13–19]. We will develop and deliver bespoke communication training to optimise surgeons’ communication about the trial, and families’ experiences of being approached. We will identify potential recruitment issues by monitoring recruitment at all sites and request sites to audio-record consultations. Researchers will review consultation recordings to inform bespoke trial communication training, which we will provide to all sites on an ongoing basis during the internal pilot.

#### Method

The communication sub-study team will provide sites with encrypted audio-recording devices. During the internal pilot, surgeons and research nurses (recruiters) will routinely seek verbal permission to audio-record consultations with families during which: CONTRACT 2 is discussed before consent is sought, consent is obtained and the family is informed of their treatment allocations. Recruiters will briefly outline the purpose of audio-recording the consultation and record the consultations if permission is granted. At the end of the consultation or at the point of taking consent for the trial, recruiters will obtain written consent for the audio-recording to be used in the sub-study. If the family declines to provide written consent, the audio-recording will be deleted. If the family provides written consent the audio recording will be transferred to the researchers via a secure encrypted system. A member of staff will also obtain written consent and complete a proforma that will capture fields providing details of the consultation, such as the name of recruiter, date of the consultation, patient age, RCT participation status and treatment allocation. Audio recording will be transcribed. The communication sub-study research team will listen to and review samples of recruitment consultations to inform bespoke feedback for each site.

#### Plan for bespoke training

Before trial recruitment starts, the communication sub-study team will deliver an initial trial communication group training session to optimise surgeon and research nurse communication with families about the trial. All recruiters who will be involved in recruiting to the trial will be invited to attend the event. This session will be based on the successful QUINTET programme [32] and will also incorporate key findings from the CONTRACT Communication Study based on our feasibility study [5, 13–19]. Training will cover key themes, such as avoiding misinterpreted or misunderstood terms, exploring family treatment preferences and highlighting families’ frequently asked questions.

We will also develop and disseminate a package of resources and support materials informed by recruiter feedback in our previous work [16] and in consultation with site staff. This includes a demonstration video that incorporates key learning points from our feasibility study [5, 13–19] and a ‘hints and tips’ handout. The ‘hints and tips’ handout will be regularly updated and circulated during the internal pilot stage, in response to ongoing review of the RCT consultations.

During the pilot stage, we will identify and offer additional support refresher training to sites that might benefit from it (e.g. those with lower approach/recruitment rates or those that have encountered challenges in discussing the trial in a balanced way with families). This will involve reviewing recruitment consultations and visiting such sites to support the team (or deliver training via video call if more appropriate) during months 3–9 of the pilot stage.

## Assignment of interventions: allocation

### Sequence generation {16a}

Patients enrolled in the trial are randomised to groups (1:1 ratio) online allowing instant assignment to treatment group 24 h per day.

Minimisation is used to ensure similarity between the groups in factors that may affect diagnostic accuracy and outcome of treatment using the following criteria:

(i) Age: 4–8 years; 9–15 years
(ii) Sex: Male; Female
(iii) Duration of symptoms (onset of pain to recruitment into study): < 48 h; ≥ 48 h
(iv) Centre

### Concealment mechanism {16b}

The online randomisation system allows complete pre-randomisation concealment of treatment allocation.

### Implementation {16c}

Once informed consent is received and eligibility for the trial is confirmed, the patient is enrolled in the trial and randomised to a treatment group (1:1 ratio) via an independent, web-based system (TENALEA) operating 24/7. The treatment group allocation will be instantly revealed to both the patient/parent and the clinical team.

## Assignment of interventions: blinding

### Who will be blinded {17a}

Due to the nature of the interventions in this trial, there will be no blinding of participants or investigators.

### Procedure for unblinding if needed {17b}

The trial design is open-label, therefore there is no unblinding procedure.

## Data collection and management

### Plans for assessment and collection of outcomes {18a}

Data will be collected at baseline by clinical teams, during hospital stay by research nurses and during follow-up period directly from parents via smartphone App or paper card at weeks 1–4 and by research nurses in other time-points. All data will be collected prospectively to ensure high accuracy and entered into an online secure trial database (Medidata) as soon as is practically possible after collection. Sites will be trained in all aspects of trial delivery including data collection in site initiation visits.

### Plans to promote participant retention and complete follow-up {18b}

Patients completing all follow up appointments and questionnaires will be offered a gift voucher (via email) as a thank you for taking part in the trial. Participants are informed of this in the trial information sheet when considering their participation in the trial.

Where possible, patients who have withdrawn from their allocated treatment will remain in follow-up as per the trial schedule. If patients additionally withdraw consent for this, they will revert to standard clinical care as deemed by the clinician responsible. It would remain useful for the trial team to continue to collect standard follow-up data and, unless the patient explicitly states otherwise, follow-up data will continue to be collected.

### Data management {19}

Participant data will be entered remotely at site and retained in accordance with the current Data Protection Regulations. The PI is responsible for ensuring the accuracy, completeness and timeliness of the data entered.

The participant data is pseudo-anonymised by assigning each participant a participant identifier code which is used to identify the participant during the trial and for any participant-specific clarification between the clinical trial unit and site. The site retains a participant identification code list which is only available to site staff.

### Confidentiality {27}

The confidentiality of participants taking part in the trial will be preserved. Investigators will ensure that participant’s anonymity is maintained. Identification codes rather than names will be used for all trial activities for all participants.

Any data collected as part of the trial will be securely stored in line with the Data Protection Act and General Data Protection Regulation.

### Plans for collection, laboratory evaluation and storage of biological specimens for genetic or molecular analysis in this trial/future use {33}

Not applicable for this trial, no samples collected.

## Statistical methods

### Statistical methods for primary and secondary outcomes {20a}

The analysis of the primary outcome (treatment success at 1 year) will be based on an intention-to-treat (ITT) population, which includes all children who have been randomised to a treatment arm (non-operative treatment pathway or appendicectomy), regardless of compliance or treatment received, and have completed data for the outcome and timepoint being analysed. Primary analysis will be by a mixed effects logistic regression model controlling for the minimisation factors with age, sex and onset of pain duration as fixed effects, each with two levels, and study site (10 or more levels) as a random effect. It is possible that controlling for study site will not be possible due to stability of the statistical model and this will be explored. Analysis will produce the absolute risk difference between the two treatment arms with a one-sided 95% confidence interval applying bootstrapping, which can then be assessed against the pre-specified non-inferiority margin of 20%. The number and percentage of children meeting the definition of treatment success at 1 year following randomisation will also be presented.

Individual outcomes that contribute to the composite primary outcome will be presented solely as descriptive statistics within the treatment pathway to which they apply (e.g. recurrent appendicitis is not relevant to the appendicectomy treatment arm). Secondary outcomes that apply to both treatment pathways will be analysed in superiority comparisons between the treatment pathways in ITT analyses, with the exception of adverse events (AEs) for which only descriptive statistics will be provided.

Further details of this, and methods for analysis of secondary outcomes, will be documented in a Statistical Analysis Plan. All analyses will be performed using STATA 19 [33].

### Interim analyses {21b}

No interim analyses are planned for this trial. Internal pilot data will be analysed after 12 months from the start of site set-up to assess trial and recruitment progress. Number of sites (i) in set-up and (ii) open to recruitment will be assessed. Overall recruitment and within-site recruitment will be assessed.

### Methods for additional analyses (e.g. subgroup analyses) {20b}

Sub-group analyses exploring study recruitment and outcomes in specialist paediatric centre versus DGH recruiting sites will be conducted. Subgroup analysis exploring outcomes from laparoscopic versus open procedures was considered but given the likely lack of open procedures may not be possible or meaningful.

#### Health economic analysis

Overall, the economic evaluation will report the long-term results from the CEA at 1 year using the primary outcome, reporting cost per successfully treated child. The CUA will report the short to medium-term cost per QALY gained at 6 weeks, when full recovery for both arms is expected. Extending the time to 1 year risks diluting the actual difference between the two treatment arms in QALY terms, and it is important to show the short-term implication in QALY terms. However, sensitivity analysis will explore long-term implications by incorporating recurrence of appendicitis, both in terms of increased costs and reduced QALYS. The potential of bias will also be explored using sensitivity analysis if increased cross-over between arms is observed. As part of the economic analysis, we will also report the economic implications for the NHS in terms of defining tariff for the non-operative treatment option and reporting on the economic impact this might have to the NHS. We will develop a full Health Economics Analysis Plan.

### Methods in analysis to handle protocol non-adherence and any statistical methods to handle missing data {20c}

The primary analysis will compare the overall ‘non-operative treatment pathway’ versus ‘appendicectomy’ and will take into account whether the initial non-operative approach was successful or not by designating per-protocol treatment switches to appendicectomy as treatment failure. This analysis, based on a comparison of two treatment pathways rather than non-operative treatment versus appendicectomy, reflects the proposed clinical pathway under investigation. It also accounts for treatment switches due to non-operative treatment failure by designating these as a treatment failure at 1 year (the primary outcome). If the non-operative treatment pathway is inferior to appendicectomy, this will be reflected in the numbers of patients who switch to appendicectomy and this will be reflected in the analysis proposed.

The per-protocol analysis will be a secondary analysis to explore the effect of non-adherence to allocated treatment (which are most likely to be due to parents’, children’s or clinicians’ desire to revert to either appendicectomy or non-operative treatment ahead of the trial schedule).

Missing data patterns will be explored to investigate mechanisms of missingness. We will consider the impact of any missing data on the primary outcome, such as sensitivity analyses.

### Plans to give access to the full protocol, participant level-data and statistical code {31c}

Southampton Clinical Trials Unit (SCTU) is committed to the responsible sharing of clinical study data and samples with the wider research community. Data and sample access is administered through the SCTU Data and Sample Release Committee, who will consider requests once the final analysis has been published. All data and appropriate documentation will be stored for a minimum of 10 years after the completion of the trial.

## Oversight and monitoring

### Composition of the coordinating centre and trial steering committee {5d}

SCTU provides day-to-day organisational support for the trial along with the Chief Investigator. A Trial Management Group (TMG) comprised of the core trial team and all co-applicants meet once per month. An independent TSC meets at least once a year. The role of the TSC is to provide oversight for the trial and advice on all aspects of the trial through its independent Chair to the TMG, Sponsor, Funder and SCTU.

### Composition of the data monitoring committee, its role and reporting structure {21a}

The Data Monitoring Committee (DMC) meets at least once a year and its role is to safeguard the interests of trial participants, monitor the main outcome measures including safety and efficacy and monitor the overall conduct of the trial. The DMC will receive and review information on the progress and data relating to this trial and provide advice on the conduct of the trial to the TSC. The DMC will inform the Chair of the TSC if, in their view, the results are likely to convince a broad range of clinicians, including those supporting the trial and the general clinical community, that, on balance, one trial arm is clearly indicated or contraindicated for all participants or a particular category of participants, and there is a reasonable expectation that this new evidence would materially influence participant management. The DMC finalised its reporting structure at its first meeting on 8-Dec-2022 and agreed a charter.

### Adverse event reporting and harms {22}

Adverse event (AE) reporting is managed on the trial database and reported to the trial team within SCTU.

Of AEs deemed to be non-serious, only those considered related to the trial interventions (rather than solely related to the appendicitis itself) need to be recorded. However, the following expected AEs do not require recording if they are not serious: (i) fever, (ii) vomiting, (iii) diarrhoea and (iv) non-recurrent abdominal pain.

For this trial, only serious adverse events (SAEs) deemed related to the trial intervention and not in the list of exceptions or list of expected AEs need to be reported to the SCTU as SAEs. The following SAEs do not require reporting to SCTU: (i) prolonged hospital stay due to treatment of appendicitis; (ii) re-admission to hospital for complication of either treatment and/or appendicitis; (iii) admission to hospital for treatment of recurrent appendicitis; (iv) hospitalisations for elective treatment of a pre-existing condition; and (v) hospitalisations for an unrelated condition. These events (if deemed trial-related) should be reported to SCTU as AEs.

### Frequency and plans for auditing trial conduct {23}

On receipt of a written request from SCTU, the PI will allow the SCTU direct access to relevant source documentation for verification of data entered onto the eCRF (taking into account data protection regulations). Access should also be given to trial staff and departments (e.g. pharmacy).

The participants’ medical records and other relevant data may also be reviewed by appropriate qualified personnel independent from the SCTU appointed to audit the study, including representatives of the Competent Authority. Details will remain confidential and participants’ names will not be recorded outside the trial site without informed consent.

### Plans for communicating important protocol amendments to relevant parties (e.g., trial participants, ethics committees) {25}

Protocol amendments will be submitted via IRAS for REC/HRA approval where necessary and distributed to all trial sites when these have been approved.

### Dissemination plans {31a}

If they consent to receiving the information, patients or parents will be notified of the results of the trial via the site where they were recruited. The data will be published in a peer-reviewed journal and available in the public domain. Our Study Specific Advisory Group (SSAG) will write a report for participants to ensure the language is appropriate and accessible.

## Discussion

Although there are an increasing number of reports concerning the use of non-operative management of uncomplicated acute appendicitis in children, and a marked increase in the use of this intervention in many jurisdictions during the recent SARS-CoV-2 (COVID-19) pandemic [34, 35], there are very few studies that have undertaken a comparative analysis of the clinical effectiveness of non-operative treatment pathway when compared to appendicectomy. We believe that such comparative analysis is essential to guide future healthcare decisions by clinicians, patients and families.

We acknowledge that Svensson and colleagues [10] have undertaken a small pilot RCT comparing these two treatments, and recently, since the initiation of this trial, two other groups [36, 37] have published findings of larger RCTs (one in Australia, one multinational). Additionally, Minneci and colleagues [11] have reported a large series of children with uncomplicated appendicitis from the USA (*n* = 1068) of whom 370 were treated non-operatively. Using a propensity score matched analysis, they assessed the impact of treatment approach on outcomes, but they did not use randomisation to assign patients to treatment arms. The two larger trials [36, 37], with a similar non-inferiority randomised design to this study, concluded that non-operative treatment was inferior to appendicectomy based on a non-inferiority margin of 20%. Despite this, we believe there is benefit in further comparative effectiveness research in this field for a number of reasons. Firstly, there are subtle differences between our protocol and others—the use of the complicated appendicitis score and a minimum of 24 h of non-operative treatment may both independently increase the likelihood of success of non-operative treatment. Secondly, we believe a further RCT is justified that is specific to the UK setting to provide an assessment of these healthcare technologies in the UK. This is particularly true for a comparative health economic analysis, since the NHS is a unique healthcare economic environment. We also note some differences in the management of appendicitis in the UK compared to other countries that reinforce the rationale for a trial within the UK. Most notably, in the UK, there is only limited use of diagnostic radiology, a relatively high negative appendicectomy rate and limited use of same day discharge after appendicectomy. Since all these factors may influence the comparative effectiveness and cost effectiveness of treatment, we believe a further RCT in the UK is justified. Finally, we note that, although analysis of the primary outcome is important, there is also significant value in understanding the impact of different management strategies on many of the secondary outcomes, since these data are likely to inform shared decision-making models with families going forwards. Understanding these accurately in the UK healthcare setting will result in the most value for NHS patients in the future.

Prior to our feasibility study [5, 13–19], we identified that recruitment may be a challenge for this comparative effectiveness research. We will be reliant on clinical teams (rather than research nurses) to screen potential participants during an emergency admission to hospital, potentially at all hours of the day and all days of the week. In an attempt to optimise recruitment in our feasibility study, we implemented a program of detailed site training, focusing not only on trial processes, but in particular on the way in which site teams communicate with potential participants and their families about the trial [5, 13–19]. Starting with indicative training based on previous research, our communication sub-study alongside the feasibility study [16, 17] allowed us to develop a bespoke training program for site teams specific to this trial. In this phase III RCT, we base initial site training on the findings of our feasibility study [5, 13–19], and also deliver a communication sub-study during the pilot stage of our RCT. We anticipate that this will be of particular importance at sites not involved in the feasibility study.

Selecting the most appropriate primary outcome for any RCT is important. It has an impact on how the trial is interpreted, how likely the findings are to change clinical practice and, in conjunction with the proposed statistical analysis, guides the sample size requirement. During our feasibility study [5, 13–19], we focused on defining the primary outcome for this trial based on feedback from patients, families and surgeons. For this trial, we defined a composite primary outcome, because it encompasses aspects that are important to all these stakeholders in determining whether treatment has been a success. Furthermore, each component of the composite has similar impact on the patient/family, since the occurrence of any will result in unplanned general anaesthesia. This threshold of requiring general anaesthesia is something that patients and families have told us is important to them.

The trial will be analysed using a non-inferiority analysis and a 20% non-inferiority margin. This margin was identified as being acceptable during our feasibility study following discussions with families and surgeons [5, 13–19]. Our non-inferiority approach is supported by the fact that patients and families recognize that non-operative treatment may be slightly less effective (inferior) to appendicectomy, yet they are willing to accept this in order to realise the potential benefits of non-operative treatment. Our feasibility study identified that surgeons too are willing to accept this but their threshold for non-inferiority is more conservative (nearer to zero) than that of patients and families [5, 13–19]. The 20% margin is therefore something of a compromise in order to suit all stakeholders. In addition to reporting the primary outcome for each treatment arm, we will report all components of our primary outcome individually as well as a range of secondary outcomes. We anticipate that this breadth and depth of data will likely be of great interest to patients, families and clinicians when making treatment decisions for each individual patient in the future.

In keeping with the concept that we aim to test the hypothesis that non-operative treatment pathway is non-inferior to appendicectomy, and assuming an existing non-zero difference between them, we will use a one-sided 5% significance level in our primary analysis. We note some controversy in the literature regarding the use of a one- or two-sided 5% significance level in non-inferiority trials and have considered the most suitable approach for this specific trial in some detail. Based on existing data [8–10], the overall direction of effect of the composite primary outcome will be in favour of appendicectomy. The size of that effect will be determined by the contribution of each individual component to generate a composite effect size. At present, there are no published data to suggest that the overall direction of the effect of the composite primary outcome will be in favour of non-operative treatment pathway. That is, based on existing data [8–10], there is no justification for exploring whether non-operative treatment pathway is superior to appendicectomy for this primary outcome. Furthermore, according to the recent review of non-inferiority trials in the UK [38], although it is less common to have a non-zero assumed difference (7/114 reviewed trials), 5/7 hypothesised that the intervention could only be worse, of which 3 used the statistical approach of one-sided 5% significance level [39–41]. On this basis, we intend to use a one-sided 5% significance level in our non-inferiority analysis of the primary outcome.

## Trial status

The current protocol is Version 8.0 from 30 October 2024. Recruitment began on 3 March 2022. Recruitment will complete approximately March 2026.

## Data Availability

Data will be available for sharing after the primary analysis is completed. Researchers interested in our data will be asked to complete the Request for Data Sharing form template located on the SCTU web-site, www.southampton.ac.uk/ctu to provide a brief research proposal on how they wish to use the data. It will include the objectives, what data are requested, timelines for use, intellectual property and publication rights, data release definition in the contract and participant informed consent, etc. If considered necessary, a Data Sharing Agreement from Sponsor may be required.

## Abbreviations

AE: Adverse event
CEA: Cost-effectiveness analysis
CHU-9D: Child Health Utility 9 Dimensions instrument
CONSORT: Consolidated Standards of Reporting Trials
CONTRACT: CONservative TReatment of Appendicitis in Children–randomised controlled Trial
COS: Core outcome set
CRN: Clinical Research Network
CRP: C-reactive protein
CSRI: Client Service Receipt Inventory
CUA: Cost-utility analysis
DMC: Data Monitoring Committee
HE: Health economics
HRA: Health Research Authority
HRQoL: Health-related quality of life instrument
IV: Intravenous
IRAS: Integrated Research Application System
NBM: Nil by mouth
NHS: National Health Service
NIHR: National Institute for Health Research
PLICS: Patient Level Information and Costing Systems
PPI: Patient and public involvement
PSSRU: Personal Social Services Research Unit
QALY: Quality Adjusted Life Year
QUINTET: Qualitative Research Integrated within Trials programme
RCT: Randomised controlled trial REC: Research Ethics Committee
REC: Research Ethics Committee
SAE: Serious adverse event
SARS‑CoV‑2: Severe acute respiratory syndrome coronavirus 2
SCTU: Southampton Clinical Trials Unit
SPIRIT: Standard Protocol Items Recommendations for Interventional Trials
SSAG: Study-specific advisory group
TENALEA: Trans European Network for Clinical Trials Services
TM: Trial manager
TMG: Trial Management Group
TSC: Trial teering committee
UK: United Kingdom
USA: United States of America
WBC: White Blood Cell
YPAG: Young Person’s Advisory Group
